# Factors affecting improvement after intravenous administration of recombinant tissue plasminogen activator (rtPA) among patients with acute ischemic stroke: A historical cohort study

**DOI:** 10.22088/cjim.15.2.251

**Published:** 2024

**Authors:** Seyed Mohammad Masood Hojjati, Amir Hossein Hasanpour, Hoda Shirafkan, Hoda Naghshineh, Ali Alizadeh Khatir, Payam Saadat, Fatemeh Sahebian, Rahele Mehraeen

**Affiliations:** 1 Department of Neurology, Babol University of Medical Sciences, Ganjafrooz Street, Babol, Iran; 2 Student Research Committee, Babol University of Medical Sciences, Ganjafrooz Street, Babol, Iran; 3 Social Determinants of Health Research c=Center, Health Research Institute, Babol University of Medical Sciences, Ganjafrooz Street, Babol, Iran; 4 Mobility Impairment Research Centre, Health Research Institute Clinical Research Development Unit of Rouhani Hospital, Babol University of Medical Sciences, Babol, Iran; 5 Ayatollah Rouhani Hospital, Babol University of Medical Sciences, Ganjafrooz Street, Babol, Iran; 6 Department of Radiology, Shahid Beheshti Hospital, Babol University of Medical Sciences, Ganjafrooz Street, Babol, Iran

**Keywords:** Ischemic stroke, Recombinant tissue plasminogen activator (rtPA), Thrombolytic therapy, Prognosis

## Abstract

**Background:**

One of the most effective treatments for patients with acute ischemic stroke (AIS) is intravenous recombinant tissue plasminogen activator (rtPA) which can minimize mortality and morbidities. In this historical cohort study, we investigate the factors affecting clinical outcomes after IV thrombolysis for AIS.

**Methods:**

We included 87 patients with acute ischemic stroke who were treated with rtPA between 2015 and 2019. Demographic and clinical data were recorded. The National Institutes of Health Stroke Scale (NIHSS) was used to assess the clinical outcomes.

**Results:**

36 patients showed lack of improvement at discharge. In unadjusted model, hypercholesterolemia was the only predictor of lack of improvement (P= 0.043; OR=0.304; CI= 0.096-0.963). After adjusting, hypertension (P= 0.018; OR= 0.18; CI= 0.043-0.749) and hypercholesterolemia (P= 0.008; OR= 8.68; CI= 1.773-42.54) were independent determinants of lack of clinical response. To evaluate risk factors in association with the duration of hospitalization, we found variables which lengthened hospitalization span including; age over 60 years (HR= 0.42 P= 0.002), hypercholesterolemia (HR= 2.19 P= 0.031), Angiotensin-converting enzyme (ACE) Inhibitors consumption (HR= 1.87 P= 0.022), and type of infarction (non-lacunar) (HR= 0.51 P= 0.026). Results indicated no considerable relationship between dose of rtPA and the appropriate response to treatment (OR=8.686 P= 0.324).

**Conclusion:**

The closer dose of rtPA goes up to standard range, the more chance of improvement will gain without increasing the risk of symptomatic intra-cerebral hemorrhage (SICH). Determining factors involved in intravenous reperfusion outcomes help physicians to identify the patients who benefit the most from rtPA.

Ischemic stroke is one of the leading causes of disabilities and death around the world. Stroke disabilities can have enormous social and financial impacts. Since life expectancy is increasing in communities, an increase in stroke incidence is also anticipated (1). Intravenous rtPA is one of the most effective treatments for acute ischemic stroke which discourage mortality and morbidities. The best time for thrombolytic therapy is within the 3 to 4.5 hours after the ischemic event (1-4). They can decline problems and disabilities in this period with a considerable possibility (5). However, the earlier we start thrombolytic therapy, the better outcomes will be (6).

other studies show that improving at least 3 points in NIHSS after 15 minutes and at least 5 points after 30 minutes is related with better results (10).

Incidence age under 60, blood glucose under eight mmol/L, mild to moderate stroke, and significant status enhancement after 24 hours are also predictors of an appropriate prognosis (11). This study aimed to assess the effect of intravenous recombinant tissue plasminogen activator (IV-TPA) on improvements and duration of hospitalization in patients with acute ischemic stroke, and the related factors. 

## Methods

Included patients was admitted to Ayatollah Rouhani Hospital of Babol, northern Iran, with clinical symptoms of acute ischemic stroke between 2015 and 2019. Patients with suspected ischemic stroke were seen within 4.5 hours of symptom onset at the Ayatollah Rouhani Hospital emergency room.

Relevant data were recorded on admission, and then during evaluation as well as treatment. Demographic information, including age, sex, past medical history of hypertension, diabetes mellitus, drug history, blood chemistry test, and imaging studies, whether brain computed tomography scan (CT scan) or magnetic resonance imaging (MRI), as well as NIHSS on admission and discharge were registered. Registrars were trained nurses and certified in recording NIHSS. According to the national American Heart Association/ American Stroke Association (AHA/ASA) guidelines, time from symptom presentation to the patient’s arrival in the emergency department and from the arrival in the emergency department to the start of intravenous TPA was also registered (door to needle time). 

The treatment decision was made by a neurologist based on the National Institute of Neurological Disorders and Stroke (NINDS) study criteria (12). The informed contest was obtained from the patients or their relatives. Having an ischemic stroke with a defined time, measurable impact on NIHSS and a confirmatory CT-scan were considered as the major criteria according to guidelines (12). Due to changes in guidelines in recent years, patients with seizures at the onset of stroke or impairment symptoms such as aphasia, that were not previously candidates for treatment, due to low NIHSS, also underwent intravenous thrombolysis.

A routine assessment was done to determine the mechanism of stroke and its subtypes( based on TOAST) in all patients, which included examinations, echocardiography, echocardiography (ECG), Brain MRI, non- invasive extra and intra- cranial carotid, and vertebral arteries imaging, including ultrasound, or magnetic resonance angiography (MRA), or computed tomography angiography (CTA) according to the available guidelines (12, 13).

Based on TOAST study, the patients are diagnosed with small-vessel occlusion (acunear infarct), if they have one of traditional clinical lacunar syndromes as well as a normal CT scan/ MRI or relevant rain stem or subcortical hemispheric lesion with diameter less than 15 mm. They should not have any evidence of cerebral cortical involvement. A history of hypertension or diabetes would support this diagnosis. Patients over 80 years of age and those treated with an anticoagulant (without considering PT, PTT, INR) were excluded from the study. Definition of Variables: Demographic information, medical history, medication use, and other clinical information were received from the patient or their companions upon admission. The stroke subtype was diagnosed based on symptoms, physical examination, and neuroimaging. The initial CT-scan and subsequent imaging, if performed, were observed and recorded by the patient's neurologist or radiologist. However, they were re-read during the study by a neurologist who was blind to the patient's treatment outcome. 

Laboratory studies were recorded in the database after receiving the answer, and other findings. The relationship between lack of recovery until discharge, the length of hospital stay, and variables including independent demographic, clinical, and hemodynamic variables were assessed. In our analysis, independent variables included age, sex, weight, hypertension, diabetes, cardiovascular disease, hypercholesterolemia, previous cerebrovascular accident (CVA), smoking, alcohol, high blood sugar, a total dose of rtPA, imaging findings (cortical involvement, new infarction, extent of ischemic changes, and hemorrhagic transformation), Stroke type, WBC count, hematocrit, creatinine, baseline NIHSS, time from stroke onset to treatment (rtPA), duration of hospitalization, the side of the defect.

Outcome measures: We defined the lack of improvement by NIHSS at 3-point decrease or equal stage of the baseline on-discharge (13, 14). All patients were followed-up during the hospitalization and NIHSS was measured alongside lab data on two points; admission and discharge. 

Statistical Analysis: Descriptive results were shown as mean (+/- standard deviation- SD) and frequency (percentage %) for quantitative and qualitative variables, respectively. To evaluate the baseline information between two groups of improvement and lack of improvement, we used independent t-test, chi-square, or Fisher’s exact test. Two analyses were performed. The first one was a multivariable logistic regression to assess the association between variables and lack of improvement according to 3-point diminish in NIHSS. We chose the factors with a considerable effect size or a significant p- value in univariate analysis. Then, entered them into final model. Adjusted odds ratio (AOR) and their 95% confidence interval (95%) were reported. In second analysis, we used Cox regression, Kaplan-Meier (log-rank test) to assess factors involved in the length of hospitalization. Improvement at discharge time was considered as the event of interest. Analysis was performed using Statistical Package for the Social Sciences (SPSS) software (Version 16.0), and p-value < 0.05 was considered significant.

## Results

From March 2015 until March 2019, 1828 patients with a cerebrovascular accident were admitted to Ayatollah Rouhani Hospital. 157 of them were confirmed with ischemic stroke and consent to treatment with IV-rtPA as well, but among them, only 87 (55.4%) patients were eligible for that. Four patients died during hospitalization. One of them caused by the intra-cranial hemorrhage [ICH] following rtPA administration.

The mean age was 62.1 (range 27-81; SD=11.70) and the male/female ratio was 47/40. Demographic information and distribution of risk factors are shown in table 1. Aphasia was the most and imbalance was the least common presenting symptom. 

On-admission, Symptom’s data are shown in table 1. The mean NIHSS before treatment was 10.8 (range, 2 to 24). 37 (42 %) patients experienced lack of improvement during hospitalization. Median NIHSS change was 5 in improvement and 2 in the other groups.

The mean time of stroke onset to hospital admission was 73.9 minutes (range 13-190; SD=43.74), and the meantime of admission to treatment initiation (door-to-needle time) was 48.4 minutes (range 3-135; SD=28.92). In an overall view, the mean stroke onset to treatment initiation was 119.3 minutes (range 28-290; SD=52.73). Besides, 28.7 % of patients were treated within 90 minutes, and 88.5 % within 180 minutes from stroke onset. Hemorrhage occurred in three cases (3.4 %) and one of them passed away during treatment. According to CT scan findings, cortex involvement occurred in 25.3 % of patients. In 12.6 % of cases, no abnormality was found in neuroimaging studies. Neuroimaging data are shown in table.1

**Table 1:** Demographic and baseline information and distribution of patients with AIS

		Overall (n=87)	With improvement (n=56)	Without improvement (n=25)	P-value
Age, mean (±SD), y		62.1 (±11.7)	61.32(±11.49)	64.84(±11.21)	0.203^ a^
Age under 60 y		31 (35.6 %)	24(42.9%)	4(16.0%)	0.019^ b^
Male		46 (52.9 %)	32(57.1%)	14(56.0%)	0.924^ b^
Weight, mean (±SD)		74.9 (±8.2)	74.53(±8.69)	76.92(±7.42)	0.238^a^
Risk factors	**Hypertension**	49 (56.3 %)	34(60.7%)	13(52.0%)	0.463^ b^
	**Diabetes mellitus**	34 (39.1 %)	24(42.9%)	8(32.0%)	0.356^ b^
	**Cardiovascular disease**	8 (9.2 %)	4(7.1%)	4(16.0%)	0.207^ b^
	**Hypercholesterolemia**	17 (19.5 %)	7(12.5%)	8(32.0%)	0.037^ b^
	**History of stroke**	8 (9.2 %)	5(8.9%)	3(12.0%)	0.669^ b^
	**Current Smoking**	11 (12.6 %)	1(4.0%)	8(14.3%)	0.174 ^b^
	**Atrial fibrillation**	9 (10.3 %)	5(8.9%)	4(16.0%)	0.350^ b^
	**Prior TIA**	8 (9.2 %)	5(8.9%)	3(12.0%)	0.669^ b^
	**Prior CVA**	23 (26.4 %)	15(26.8%)	7(28.0%)	0.910^ b^
Previous medication use	**ACEI**	31 (35.6 %)	16(28.6%)	13(52.0%)	0.042^ b^
	**Aspirin**	27 (31 %)	19(33.9%)	7(28.0%)	0.598^ b^
	**Statin**	28 (32.2 %)	21(37.5%)	6(24.0%)	0.234^ b^

a: independent t-test; b: chi-square test; c: Fisher exact test. Significant results are shown as bold text.

High cholesterol level (P=0.043; OR=0.304; CI=0.096-0.963) was the only variable substantially related with the lack of improvement. No significant association was found between other demographic data or risk factors with lack of improvement. Logistic analysis did not reveal ga meaningful relationship between dose of TPA and lack of improvement, but indicated a high odd ratio. Due to strict criteria, large studies are needed to discover the exact relationship of these variables, as it mentioned in discussion. Complete logistic analysis data is shown in table 2. There was no significant association between base NIHSS and the appropriate response (admission NIHSS – discharge NIHSS≥3) (P=0.15). Also, patients with on-admission NIHSS less than 15 had a 54% chance of appropriate response (P= 0.157; OR=0.46). In univariate logistic analysis, hypercholesterolemia (P= 0.043) was an independent predictor of lack of improvement. However, in multivariable logistic regression, hypertension (P= 0.018) and hypercholesterolemia (P= 0.008) were predictors of it. Furthermore, with each unit increase in rtPA dose (my/ kg), the chance of improvement surged 10-fold (OR=10.948). Even though 27 patients with normal brain CT scans had appropriate responses and only 7 of them were categorized as lack of improvement, which was noted as a significant finding. 

**Figure 1:**
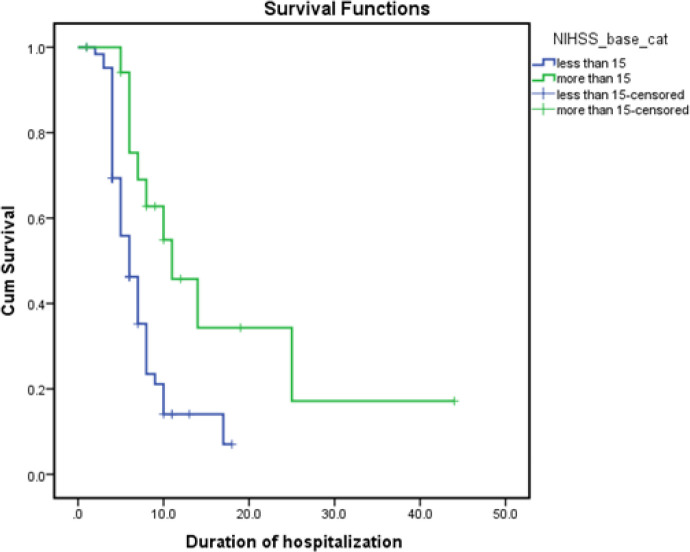
Survival graph of time duration until improvement based-on-admission NIHSS (less than 15 vs. more than 15)

Length of hospitalization: We used Cox regression to evaluate factors affecting the time to recovery (period of hospitalization) that are including ages less than 60 years (HR= 0.42 P= 0.002), hypercholesterolemia (HR=2.19 P= 0.031), ACE Inhibitors consumption (HR=1.87, P= 0.022), and type of infarction (non-lacunar; HR=0.51, P= 0.026 and lacunar; HR= 1.12, P= 0.815, reference category = normal). Also, baseline NIHSS score more than 15 illustrated a significant effect on extension of hospitalization (HR= 2.68) P= 0.003). Median hospitalization period for patients with base NIHSS more than 15 was 11 days, while patients with less than 15 experienced a median hospital stay time of 6 days (fig.1). In addition, increasing the dosage of rtPA reduced the hazard of inappropriate response by 8% (HR=0.92, P = 0.946). There was no significant relationship between time of duration stroke onset to treatment initiation and hospitalization duration (HR= 1.01, P=0.974). 

**Table 2:** Clinical characteristics of patients with AIS on admission

			Overall (n=87)	With improvement (n=56)	Without improvement (n=25)	P-value
Chemistry levels at admission, mean (±SD)	**Glucose, mg/dL**		147.4 (±71.3)	146.61(±71.95)	151.84(±78.53)	0.770^ a^
	**Creatinine, ** μmol/L		1.22 (±0.98)	1.22(±1.19)	1.21(±0.48)	0.988^ a^
	**White blood cell count**		7845 (±1913)	7969(±1995)	7624(±1835)	0.463^ a^
	**Hematocrit, %**		37.8 (±6.8)	38.23(±7.88)	36.73(±3.82)	0.368^ a^
Treatment (IV rtPA)	**Time to treatment, min mean (±SD)**		119.2 (±53.7)	118.07(±51.04)	118.48(±56.60)	0.974^ a^
	**Total dose, mg, mean(±SD)**		52.1 (±7.5)	52.26(±8.21)	52.20(±6.14)	0.844^ a^
Symptoms on admission	**Right and left paralysis**		3 (3.4%)	3(5.5%)	0(0.0%)	0.234^ b^
	**Side of hemiplegia**	**Right**	39 (44.8%)	25(44.6%)	11(44.0%)	0.957^ b^
		**Left**	34 (39.1%)	23(41.1%)	9(36.0%)	0.666^ b^
	**Aphasia**		44 (50.6%)	28(50.0%)	14(56.0%)	0.618^ b^
	**Sensory disorder**		7 (8%)	4(7.1%)	2(8.0%)	0.892^ b^
	**Loss of consciousness**		8 (9.2%)	6(10.7%)	2(8.0%)	0.705^ b^
	**Vertigo**		5 (5.7%)	3(5.4%)	2(8.0%)	0.648^ b^
	**Imbalance**		2 (2.3%)	1(1.8%)	1(4.0%)	0.562^ b^
	**Dysphagia**		4 (4.6%)	3(5.4%)	1(4.0%)	0.795^ b^
	**Urinary incontinence**		3 (3.4%)	0(0.0%)	3(12.0%)	0.027^c^
SaPrimary neuroimaging findings	**Side of defect**	**Right**	16 (18.4%)	10(18.5%)	6(27.3%)	0.434^ b^
		**Left**	27 (31%)	16(29.6%)	9(40.9%)	
		**Normal**	36 (41.3%)	27(50.0%)	7(31.8%)	
		**Bilateral**	1 (1.1%)	1(1.9%)	0(0.0%)	
	**Cortex involvement**		22 (25.3)	15(27.8%)	6(27.3%)	0.753^ b^
	**Infarct type**	**Lacunar**	5 (5.7)	5(9.3%)	0(0.0%)	0.061^ b^
		**Non-lacunar**	39 (44.8)	22(40.7%)	15(68.2%)	
		**Normal**	36 (41.3)	27(50.0%)	7(31.8%)	

a: independent t-test; b: chi-square test; c: Fisher exact test., Significant results are shown as bold text.

**Table 3:** Logistic regression model for lack of improvement in patients with AIS

Risk Factors	Unadjusted Model			Adjusted Model		
	OR	95%CI	p-value	AOR	95%CI	P-value
Age	0.971	0.869-1.067	0.204			
Sex	1.372	0.405-2.711	0.924			
Wight	0.966	0.911-1.023	0.236			
Hypertension	1.485	0.593-3.718	0.399	0.18	0.043-0.749	0.018
Diabetes	1.594	0.590-4.302	0.358			
Cardiovascular disease	.396	0.052-2.999	0.37			
Hypercholesterolemia	0.304	0.096-0.963	0.043	8.68	1.773-42.54	0.008
Previous CVA	1.391	0.305-6.335	0.67			
Smoker	0.250	0.030-2.116	0.203			
High BS	0.999	0.993-1.005	0.767			
rtPA dose (mg/kg)	8.686	0.119-636.577	0.324	10.948	0.108-111.78	0.310
CT Scan finding						
Normal	0.750	0.171-3.248	0.223			
Ischemic stroke <1/3	1.501	0.262-8.38	0.255			
Ischemic stroke >1/3	0.250	0.032-2.325	0.094			
WBC count	1	1-1	0.459			
Hematocrit	1.050	0.942-1.170	0.378			
Creatinine	1.004	0.629-1.603	0.987			
Duration of hospitalization	0.928	0.846-1.019	0.117			
Side of defect (right)	1.609	0.863-3.001	0.135			
Cortex involvement	0.625	0.178-2.199	0.464			
Infarct subtype						
Lacunar	4.19	0	0.999			
Non-lacunar	0.980	0.132-1.097	0.074			

## Discussion

This study was designed to assess factors affecting clinical outcomes following rtPA administration. Results indicated that high cholesterol level and hypertension can negatively impact outcomes of AIS after rtPA administration. Also, AIS patients who received standards dose of rtPA (0.9 mg/ kg/ in comparison with low dose showed better result without any increase in SICH. 

Although timely re-perfusion is the most effective therapeutic strategy in acute ischemic stroke (15), the outcome can be influenced by numerous factors. In this study, high cholesterol level and hypertension were the variables that significantly associated with the lack of improvement. According to the previous studies, dyslipidemia can result in the formation of a non-soluble lipid-rich thrombus and consequently the enlargement of the infarcted area and hemorrhagic transformation after receiving rtPA (16). The effects of baseline systolic blood pressure and ischemic stroke have been previously discussed in numbers of studies (19, 20). Our findings regarding the effect of pre-existing or newly revealed hypertension, on improvement after thrombolytic therapy is similar to some of the previous studies. There is a U shape relationship between blood pressure and functions after ischemic stroke, with both high and low systolic blood pressure being correlated with poor outcomes. Due to high systolic blood pressure, it might encourage the risk of brain edema, intra-cranial pressure, and stroke recurrence. It can also induce oxidative stress on blood brain barrier (20). 

Furthermore, although logistic analysis did not reveal a meaningful relationship between dose of rtPA and lack of improvement in cases (P=0.310), large studies are may needed to work on it. However, with each unit (mg/kg) increase in rtPA dose up to maximum dose (0.9mg/kg for Alteplase), the chance of improvement developed 10fold (OR=10.948). As several case series with small sample size in various Asian populations comparing the safety and efficacy of the different doses of rtPA showed contradictory results. However, a large study of non-Japanese Asian patients receiving different doses of IV-tPA indicates that the standard dose of tPA (0.9 mg/kg) is not only safe, but also may have a more favorable outcome than low doses of IV-tPA (17).

Alternately, we found no association between demographic features, baseline NIHSS score, presence of vascular risk factors, and time to treatment with lack of improvement on discharge. There is no consensus in the literature regarding the effects of sex, age, and vascular risk factors on functions after tPA administration (4, 7, 11, 13, 16, 18). On the contrary, with regard to baseline NIHSS score, our findings were similar to a study evaluating clinical predictors of lack of improvement at 24 hours (14). They were not in agreement with previous studies (4, 11, 16, 18) which analyzed the improvement at 3 months, while we reported on discharge NIHSS. Furthermore, we did not find any correlation between lack of improvement and time to treatment. This can be because of receiving tPA 4.5 hours before the appearing symptoms, with mean stroke onset to treatment initiation being less than 2 hours.

The correlation between infarct subtypes or cortical involvement and the lack of improvement was not revealed, even though lacunar type of infarct negatively affected the duration of hospital stay. Although some research projects have shown that lacunar infarcts was an independent predictor of new major vascular events (19), the functions in patients with imaging-defined lacunar infarct did not differ from those with non-lacunar ones (20). Results show that, improvement following rtPA administration was not affected by the side of ischemic lesions, cortical involvement, and presentation symptoms. Based on previous studies, hemispheric involvement, lesions’ size and topography can impact stroke severity and consequently functions (21). However. it seems that intravenous thrombolysis improves functions among ischemic stroke patients regardless of their severity (22). This can also explain why while the stroke subtype (lacunar), age (>60 years old), high base NIHSS (>15) had no correlation with lack of improvement in cases, they negatively impacted the duration of hospitalization.

The main limitations of our study were the lack of follow-up evaluation within 3-6 months after stroke. We did not also evaluate improvement 24 hours following intravenous thrombolysis. Finally, our study was performed in a single centre with a small number of patients.

Our study implies that with increase dose of rtPA up to standard dose, the chance of improvement can surge without increasing the risk of SICH. Detecting effective factors is suggested to enhance the result of intravenous reperfusion, which also enables physicians to identify the patients who benefit the most from rtPA.

.
